# Toxic Leadership and Project Success: Underpinning the Role of Cronyism

**DOI:** 10.3390/bs12110427

**Published:** 2022-10-31

**Authors:** Farida Saleem, Muhammad Imran Malik, Shabir Hyder, Ambrin Perveen

**Affiliations:** 1 Department of Management, College of Business Administration, Prince Sultan University, Riyadh 11586, Saudi Arabia; 2 Department of Management Sciences, COMSATS University Islamabad, Attock Campus, Attock 43600, Pakistan

**Keywords:** cronyism, toxic leadership, project success, information technology projects, social exchange theory

## Abstract

Project success is the backbone of competitiveness and sustainability. The study aims to examine the role of cronyism in the relationship between toxic leadership and project success while taking information technology projects as the study context. Cross-sectional data (*n* = 240) was collected through closed-ended survey questionnaires to record the responses of IT project employees. The structural equation modeling (SEM) technique was used for analyzing the collected data. Results revealed a negative relationship between toxic leadership and project success, while cronyism positively and significantly mediated the relationship and converted the negative relationship between TL and PS to a positive relationship.

## 1. Introduction

The project failure rates are constantly increasing [1]. A host of factors are responsible for such failures, including inappropriate leadership styles, unsupportive culture, ill-defined tasks, inappropriate use of project management techniques, misuse of power, and so forth [2]. The project leaders play a vital role in the timely achievement of the targets [3,4,5] and vice versa [6]. Unclear findings exist in the literature underpinning the part of leaders in project success. According to Zaman et al. [6] and Zhu, Wang, Yu, Müller, and Sun [7], employees are better controlled and motivated to achieve targets under toxic leaders, whereas Saleem, Malik and Malik [8] found only two out of five dimensions of toxic leadership harmful to the performance of employees. Moreover, Saqib and Arif [9] found that toxic leaders significantly contribute to organizational learning, leading to success.

However, in general, the inappropriate leadership style, i.e., toxic leadership, is responsible for misappropriations and failures [10]. It is dysfunctional leadership behavior [11] that negatively affects the subordinate’s attitudes and behaviors and brings negativity into the workplace [12], resulting in overall poor performance [6]. Ali et al. [13] and Lipman-Blumen [14] argued that toxic leadership had become a standard part of organizational functioning. Kusy and Holloway [15] found that 64% of respondents suffered from and survived under toxic leaders. In addition, 94% reported having worked under a toxic person at some point in their careers [16]. Bell [17] also said corporate failures due to toxic leaders in action. It is also noted that one leader toxic out of every five leaders [18]. Toxic leaders are seen to negatively affect their immediate subordinates and the organization as a whole [10]. Moreover, they are involved in corruption and cronyism and support the inadequacy of others [19], having harmful effects on projects.

Cronyism, as a problem, is associated with toxic leadership. It is the use of malpractices to distribute resources and recruit inappropriate people. Cronyism, as a source of stress for employees (who are not a part of the in-group), affects their performance negatively [20]. Pelletier and Bligh [21] have investigated that 76% of top managers used cronyism as a practice for undue favoring of their subordinates. In organizational studies, little attention has been paid to cronyism, yet is much research on social networking and political connections that help to form a basic understanding of cronyism affecting organizational practices [22]. However, there has been little discussion about cronyism in the existing literature [23]. 

The project failures are generally attributed to the pure technological perspectives and not the human side [24]. However, this is only sometimes the case, as there is always a man behind the machine. It is necessary to consider the humans affecting project success because they are major contributors toward its goal’s achievement. Ramos and Mota [25] argued that the literature is deficient in the factors responsible for the success or failure of IT projects, especially human attitudes and behaviors, and needs examination. IT projects are failing at a rate of 60 to 80% every year, despite the increase in project management education [26]. Studies have shown that the role of a project leader is vital, yet is a gap in identifying the project success factors, including the leadership style of project success [5].

In this regard, the social exchange theory—SET states that environmental factors like the leadership style and the practices adopted by the leaders (cronyism) have a definite effect on employees functioning [27,28] that can ensure project success. The present study contributes to the literature in the following ways: Firstly, earlier studies have considered technological perspectives responsible for affecting projects, whereas this study examines the human factors, especially the leaders, affecting project success as the IT project management literature has stressed the importance of leadership as a critical success or failure factor. Secondly, there is much evidence of social networks and political influences on the project’s success; however, cronyism is used to develop a framework for the first time. Thirdly, the study retests the social exchange theory by developing a framework. The current study examines the relationship between toxic leadership and project success in IT projects with the mediating role of cronyism.

With this study, we contribute to the existing literature by not only examining the role of cronyism in the relationship between toxic leadership and project success in the context of IT projects while using the lens of the social exchange theory (SET) but also by analyzing the proposed model in a developing country context. Information technology organizations are the “backbone” of developing countries, yet IT projects often have poorer performance records than other projects. Therefore, there is a need to investigate the causes of IT project failure. Researchers found project leaders’ behavioral issues to be the most critical indicator of IT project failures [29]. When a leader shows favoritism towards followers, it leads to dissatisfaction among employees. Leaders showing favoritism or awarding limited promotions and selections based on subjective rather than objective standards are generally mistrusted among employees [30]. This can lead to an unfavorable situation for the organization. Similarly, when in-group biases prevail, and the emphasis is on loyalty towards leaders, cronyism is a likely outcome and significantly affects employees’ behaviors [31]. 

### 1.1. Theoretical Framework and Literature Review

The social exchange theory (SET) posits the interaction of two parties based on cost-benefit analysis to create a win-win situation [27]. During the social exchange, one has to forego something valuable, such as loyalty and conformance to the leader, to receive something in return. The SET is used to test social relationships [32], such as the relationship between the leaders and employees who try to build a win-win situation. The leader provides the resources and expects employees’ loyalty, effort, and time to achieve the goals.

Moreover, leaders play a key role by displaying authority to manage the process of work, distribution of rewards, provision of resources, and assessing the performance of employees. At the same time, the leader provides development opportunities. Good leadership can lead to higher levels of project success [33]. In a work setting, the leaders affect the behaviors and productivity of the followers towards their organization with their decisions, character, and implementation [34]. Toxic leadership is practiced in the presence of three factors, i.e., destructive leaders, susceptible followers, and conducive environments [35], also known as the toxic triangle.

**Figure 1:**
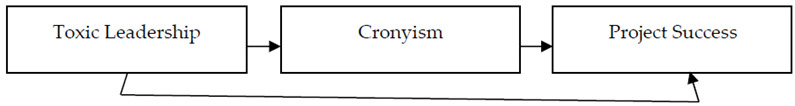
Research Framework.

One of the corollaries of toxic leadership is cronyism. In the context of SET, cronyism, as an exchange, favors awarding jobs and providing other undue advantages to friends or people closer to the leader. It also includes appointing “cronies” to positions of authority, regardless of their qualifications. Although this practice is unethical, it may increase performance [36]. The LMX theory shows that the in-group people perform more effectively than the out-group people [37] in achieving project goals. The following framework is based on the above arguments. The hypotheses are developed based on the following framework, see Figure 1.

### 1.2. Toxic Leadership and Project Success

Various understandings exist for project success; for example, projects completed within scope, time, cost, and quality are seen as successful projects [38]. Rose added that the projects completed within scope, time, cost, and quality are regarded as successful projects. Karlsen and Lereim [39] defined project success as “A finite piece of work that is finished within budget, on time, and meeting the stated specifications (i.e., value); is used by its the intended constituents; and leads directly to the organization’s improved efficiency or effectiveness.” But Müller and Jugdev [40] argued that perceptions and dimensions of success vary by personality, nationality, project type, and contract type. There are four dimensions of project success: project efficiency, impact on the customer, direct business and organizational success, and preparation for the future [41].

The factors affecting the success or failure of projects mainly include the leadership style, communication processes used, technical knowledge about project management and commitment, quality of teamwork, and so forth [40]. Leadership is vital to any work environment, especially for managing IT projects to ensure success, especially when leaders can manage people, decrease their stress levels, and manage their emotions and styles of leadership and communication [42].

Due to the complex and technical nature of IT projects, these are prone to failure, which heavily depends upon the leader and how they manage the projects [43]. Lewis [44] declared competence and working out projects as major factors for the success or failure of a project. On the contrary, it is indicated that a bad work environment created by toxic culture may harm employee performance and, thus, project success [12]. People working under such leaders tend to have poor work performance, high absenteeism, increased turnover, and so forth [45], thus harming their engagement to work [46]. Toxic leadership behavior is associated negatively with declining organizational norms and values. It puts the organization’s effectiveness at risk, which leads to losing gifted people who are not substituted quickly [47]. Toxic culture destroys a team’s morale and the entire environment of the organization [48]. When a leader attacks employees’ self-esteem, the employees experience a decrease in self-efficacy, self-worth, and low morale, resulting in performance deterioration [15] and, thus, diminished project success [49]. According to Schmidt [50], extensive research has been carried out on competencies and behaviors of leadership; however, more needs to be said about toxic leadership affecting project success. He says a toxic leader is a narcissistic, self-promoting person who adopts an unusual injurious and authoritarian supervision pattern. Toxic leadership significantly affects individuals, teams, and organizational performance [51] and plays a vital role in shaping workplace discrimination [52]. 

Traditionally, it is argued that toxic leadership is a costly phenomenon. It destroys individuals, groups and, organizations, even countries by negatively influencing the emotions and behaviors of individuals [10]. Moreover, toxic leadership destroys the organizational culture and climate, leading to low organizational commitment, low motivation of employees, low job satisfaction, decreased employee performance, and creating ever-lasting harm by violating the legitimate interests of the individuals and organization [53]. At the same time, higher turnover and reduced loyalty and commitment are observed among employees [54], which further harms the operations. It decreases employee productivity, health problems, stress, and burnout [55]. Indra Devi [10] confirmed toxic leaders as totally destructive people who drain individuals and organizations’ energy. They create a fearful atmosphere that halts smooth functioning. 

It is further noted that the greater the presence of toxic practices in the workplace, the more significant the negative impacts on an organization’s ability to retain key staff [56] due to the prevalence of job stress, workgroup cohesion, and target abuse towards peers and interpersonal deviance [46]. Toxic leaders show unethical behaviors that are directly associated with organizational corruption [57], thus harming the existence of employees. The hypothesis developed is:

**Hypothesis** **1**: *Toxic leadership negatively affects project success*.

### 1.3. Cronyism as a Mediator

Cronyism is “favoritism shown by the superior to his or her subordinate based on their relationship, rather than the latter’s capability or qualification, in exchange for the latter’s loyalty” [31]. It is important to make a distinction between the four types of unethical behavior. They are nepotism, cronyism, patronage, and favoritism. The difference is that nepotism occurs in blood relationships, while cronyism refers to recruiting employees based on friendships and patronage occurs when political or religious membership is involved. In cronyism, the cronies (long-lasting friends) are not relatives but friends and acquaintances. Both of them encompass the selection and promotion of people not based on ability and performance [58]. At the same time, favoritism refers to the endowment of special privileges to friends and colleagues in employment, profession, and personnel decisions [59].

Cronyism takes place in organizations in two ways: horizontal cronyism and vertical cronyism. Horizontal cronyism occurs among peers such as coworkers. In contrast, vertical cronyism occurs between a leader and subordinate(s), and in this type of cronyism, the leader favors the subordinate by overlooking their performance-related factors [23]. Favoritism causes damage to proficient managers in an organization and makes it difficult to find competent candidates [60]. It is well known that nepotism, a part of cronyism, brings about negative employee attitudes towards the organization and decreases their devotion to their jobs, which handicaps the productivity and success of an organization [61]. As nepotism only favors relatives in all positions, cronyism favors only companions and friends in every situation, and patronage means that the governing political party appoints its friends and relatives to only high positions, not lower positions. Cronyism destroys the importance of doing good deeds for others and entails damage to the organizational property, that is, the use of public goods for personal benefits [23].

Stress and workplace discrimination emerge from inequality and cronyism in the workplace, resulting in destructive employee work outcomes. These consequences include lower performance, declining job satisfaction, organizational commitment, stress, intention to leave, bullying, a decrease in organizational citizenship behavior, and so forth [62]. Furthermore, Shaheen, Bashir, and Khan [63] found that corporate cronyism is positively related to psychological contract breaches for the out-group people, leading to deviant workplace behaviors. Whereas Büte [64] noted that cronyism negatively affects workers’ performance and their connections in the organization suffer from discrimination, leading to low job satisfaction and commitment. 

Contrary to the above negative implications of cronyism, Azam and Qureshi [65] argued that cronyism or favoritism increased employee productivity and job satisfaction due to personal connections. Similarly, it is noted that cronyism does not always result in a lowering of organizational performance, as employees may feel forced to perform better to protect their jobs than those whom they perceive as being favored [66]. Cronyism is likely to occur when there is an overemphasis on showing loyalty and strong in-group bias or both [31]. It is noted that the Chinese and Indians show more commitment to their leaders than their organization, thus strengthening cronyism. From a developing country such as Pakistan’s perspective, cronyism is not considered destructive and illegal, as people accept such relationships in exchange for personal benefits without resisting. Therefore, cronyism positively affects employees’ satisfaction in developing countries [65].

In a study, Shabbir and Siddique [67] concluded that in developing countries, competencies and skills do not matter in the hiring process; vacancies are filled based on personal relationships. Therefore, according to Asgharian et al. [68], sociability and friendly behaviors among organizational members breed interpersonal relations rather than actual performance [69]. Leung and Bradley [70] presented the positive implications of ethical cronyism on generating performance. 

The toxic leader exhibits cronyism behaviors based on inequity and discrimination [30]. Employees close to the leader enjoy a rich atmosphere filled with opportunities, and the leader shares more leadership experience with in-group members. This is why the in-group members are more passionate about participating in complex and challenging assignments [71]. According to Lipman-Blumen [19], toxic leadership behavior involves corruption and cronyism, but this cronyism can lead to higher performance levels by employees. Similarly, collectivist societies practice cronyism because their culture is based on familial and tribal relationships [72]. Hence, based on the above discussion, the following hypothesis is developed:

**Hypothesis** **2**: *Cronyism positively mediates the relationship between toxic leadership and project success*.

## 2. Methodology

### 2.1. Sampling and Data Collection

The data were collected from male and female software and information technology project employees. The selection of software and IT project employees was due to two main reasons: First, many IT and software development companies are located in the Islamabad Capital Territory (ICT) of Pakistan, making data collection easy. Secondly, the industrial environment is very competitive due to many well-known IT and software development firms operating here. To deal with a highly competitive environment, leaders are prone to becoming toxic. 

The minimum sample size *n* = 208 was calculated using the online sample calculator “Raosoft” with a 5% margin of error, 85% CI, 50% response distribution, and a population size of 200,000 or more. The snowball sampling technique was used as it helped generate responses from a sample of 240 middle-level managers (employees). Partial least square structural equation modeling was used to test the framework.

### 2.2. Survey Instrument

Informed consent was obtained from all respondents to the survey. The consent statement “I have read, and I understand the provided information and have had the opportunity to ask questions. I understand that my participation is voluntary and that I am free to withdraw at any time, without giving a reason and without cost.” was provided to each participant of the survey before filling out the survey form. The survey included all proposed variables presented in the proposed research model. All variables were measured on a 5-point Likert scale where “strongly disagree” was 1 and “strongly agree” was scored as 5. 

#### 2.2.1. Cronyism

The term cronyism is operationalized as presented by Khatri, Tsang, and Begley [23], as it is a reciprocal exchange transaction where party “A” shows favor to party “B” based on shared membership in a social network at the expense of party “C”’s an equal or superior claim to the valued resource. The questionnaire for cronyism was adopted from Turhan [73]. There were a total of 15 items, such as; “My manager treats employees with whom he has a close personal connection with more tolerance,” “In my institution, individuals’ performance rather than their relations with the manager is taken into account when employees are rewarded,” “When resolving conflicts, my manager protects employees with whom (s)he has a closer personal connection” and so forth.

#### 2.2.2. Toxic Leadership

According to Schmidt [50], toxic leaders are narcissistic self-promoters who participate in an unusual, injurious, authoritarian supervision pattern. The questionnaire was adopted from Schmidt [50]. It had a total of 15 items, which included statements like; “My current supervisor drastically changes his/her demeanor when his/her supervisor is present,” “My current supervisor will only offer assistance to people who can help him/her get ahead,” “My current supervisor accepts credit for successes that do not belong to him/her” and so forth.

#### 2.2.3. Project Success

Project success refers to a finite piece of work completed on time, under budget, and meeting clients’ required specifications (i.e., value) used by its intended constituents. It leads directly to the organization’s improved effectiveness and efficiency [74]. The items adapted for measuring information technology project success were 10 in number, including statements such as; “The project achieved all its intended outcomes as defined by the client,” “The project made a significant and valuable contribution to organizational goals,” “The project made a significant and valuable contribution to the society” and so forth.

## 3. Data Analysis and Results

The results are divided into two sections: the demographic information of respondents and the structural equation model results. Table 1 presents the demographic data of the respondents. 

Table 1 presents the data collected from male and female IT project employees, wherein almost one-third of the respondents who took part in the survey were male. The remaining were female respondents (68.8% and 31.2%, respectively). Nearly half of the respondents (42.0%) were aged between 36 and 45 years, followed by 36.6% of people who belonged to the age group between 26 and 35 years. It is also noted that more than half of the respondents were well-qualified; they obtained master’s qualifications (65.9%), while most other employees possessed a graduate qualification (18.3%). A maximum of the respondents had a handful of experience of 1 to 5 years (38.8%), followed by people with experience of less than a year (29.6%). 

### 3.1. Measurement Model/CFA Analysis

The study used structural equation modeling using the partial least squares method (PLS-SEM). In the first stage, the assessment of the measurement model was performed, whereas the second stage involves the evaluation of the structural model by Hair, Ringle and, Sarstedt [75]. Table 2 contains information about the factor loadings and uses Composite Reliability (CR), and Average Variance Extracted (AVE) to access the reliability and validity of the constructs. Moreover, to gauge convergent validity, the authors evaluated the average variance extracted (AVE) statistics for each construct.

Table 3 presents the discriminant validity. The discriminant validity is established if the square root of constructs’ AVEs is greater than the inter-correlations of other constructs. In this study, the results of the analysis show that the square root of AVE was greater than the correlation between each pair of constructs, as shown in Table 3, thus providing evidence for discriminant validity. Table 3 reports that all the diagonal elements (square root of AVE) are greater than the off-diagonal elements [76]. Overall, the measurement model results are satisfactory and suggest that it is appropriate to proceed further with the evaluation of the structural model.

### 3.2. Structural Model Analysis

The structural model describes the relationship among the constructs examined by developing a framework and applying statistical tests [75]. The structural model depicts the relationship between the exogenous and the endogenous variables. The present study analyzed the structural model using the maximum likelihood method in SmartPLS. Table 4 presents the standardized parameters. The bootstrapping simulation was performed to confirm the significance of the hypothesis. It is noted that when the direct relationship between toxic leadership and project performance was examined, it resulted in a significant negative relationship (beta = −0.542, *p* < 0.05), but as soon as cronyism was added to the model, the relationship between toxic leadership and project success turned positive (beta = 0.634, *p* < 0.05). Upon examining the mediating effects of cronyism in the relationship between toxic leadership and project success, it was found that it significantly mediated the relationship (beta = 0.327, *p* < 0.05). It was found that cronyism plays a major role in projects, that projects are managed through developing networks and relationships and that toxic leaders try their best to attain loyalty towards themselves instead of the project. However, cronyism played a positive mediating role in converting the relationship between TL and PS into a positive one, which contradicts most of the existing literature.

## 4. Discussion 

The current study examined the relationship between toxic leadership and project success (Hypothesis 1) with the mediating role of cronyism (Hypothesis 2) in the context of information technology projects in Pakistan. The findings showed a negative relationship between TL and PS at first; the findings were supported by the existing literature that believed toxic leaders to be harmful to individuals [40,43,50] and projects [77]. Earlier studies believed that toxic leaders bring negativity that leads to project failure. They suggested that to deliver a project successfully within cost, time, and quality, there is a need to reduce toxicity [78].

The negative relationship between toxic leadership and project success is a result of distress and preference-based treatment displayed by such leaders [79]. Saleem, Malik, and Malik [8] argued that toxic leaders take credit for others’ doings and show less respect to others, thus creating an environment of mistrust. Such an environment discourages the project team members from showing commitment to their work. Moreover, toxic leaders are narcissists [80] and show unpredictable behaviors by displaying a lack of empathy toward the project team members [81]. They use the project resources to fulfill their requirements instead of achieving the project goals. They display rude behaviors and ignore others’ needs. They withhold essential project information [81] and avoid teaching others. By their toxicity, they avoid responsibility and bring others down to build themselves up. Leaders with such characteristics pay less attention to project team members’ problems, requirements, and suggestions. Under such conditions, the project team members develop lower morale and lose attention regarding the project work; thus, projects result in failures. Toxic leaders create hostile work environments [82] through their behaviors and by promoting their cronies. It creates a hostile competition environment and does not promote collaboration, leading to slow-paced project work.

Cronyism is a form of preferential treatment that leads to organizational injustice that further reduces the efficiency and effectiveness of individuals working there. Cronyism, as a negative factor adversely affects organizational performance by producing low morale, organizational inertia, and so forth [31,83]. The adverse effects of toxicity include the leader’s complicating the organizational structure by using poisoned power, reducing employee’s work efficiency and productivity through harmful behaviors such as disappointing workers, disregarding thoughts, marginalization, aggravation, emotional volatility, blaming others for personal mistakes, threatening employees’ job security and so forth and the use of cronyism [84].

The relationship between TL and PS turned positive when cronyism was added as a mediator. Cronyism proved to be a positive player instead of having a negative impact. Azam and Qureshi [65] argued that cronyism or favoritism increased employee productivity and job satisfaction within the workplace due to personal connections. This may be true for the people working in the in-group instead of the out-group. The in-group people are nearer to the leaders and managers, receiving more favors and enjoying more resources, better positions, and benefits. Emphasis on personal relationships and loyalty, to a certain degree, may be functional and does not necessarily breed cronyism. For instance, sociability, which refers to sincere friendliness among members of an organization, raises morale, fosters teamwork, and promotes creativity [31]. Cronyism may only sometimes result in lower organizational performance because employees may feel forced to perform better than those they perceive as being favored to protect their jobs [66]. At times project leaders look for the “yes men” attitude to follow the instruction as and when given to run the activities as per time and resources that may produce positive consequences. 

Moreover, in the context of collectivist societies, cronyism is considered a right to be exercised, and it is used to enhance the productivity of employees because, as they are generally hired based on personal and familial relationships, they try their best to perform well to earn their place in projects/organizations. For cronyism, it is further argued that the in-groups have higher job satisfaction and commitment than out-groups because insiders are preferred over outsiders despite having equal skills and abilities [31]. Similar leadership behaviors can be observed in the IT sector across developing countries where people feel it convenient to use informal networks as strategic success factors, especially for solving IT project-related problems.

### Managerial and Practical Implications

The study posits that the harmful practices from leaders promote work deviations, lack of commitment, and withdrawal behaviors on the part of project team members that reduce the chances of project success. Moreover, the toxicity practiced by leaders works as a medium of social exchange in projects that force team members to avoid the work environment, thus reducing the chances of success. IT projects are characterized by teamwork and close integration of activities to complete the projects in time, which is only possible through collaboration and the sharing of resources and knowledge. But with the inclusion of the cronies in the project, things become different. The negative behavior of leaders puts other employees under high pressure to compete with those cronies to retain their jobs and achieve a good position in the project. This enhances their performance, which leads to the timely achievement of the project goals. 

The literature has time and again emphasized the removal of toxicity from organizations and projects. From the results of this study, it can be understood that cronyism can be a source of diminutive toxicity. Cronies generally have easy access to project resources. Cronies may develop strong interactions with non-cronies to avoid whistle-blowing and share resources, which promotes timely goal achievement.

The toxic leader has a relationship of trust with the cronies, and the cronies likely help the leaders to achieve the targets on one side. On the other the non-cronies make efforts to retain their positions in the projects, thus working hard and completing the project goals. In this way, the toxicity depicted by the leaders turns into a healthy interaction between cronies and non-cronies.

Toxic leaders tend to be less skillful in terms of handling projects and promote cronyism. However, cronies may help them run the projects in the desired manner and help them achieve project goals. 

As an unexpected proof, toxic leadership had a positive relationship with IT project success due to the practice of cronyism. As per Hofstede, Pakistan has a collectivist culture [85] that values personal relationships and loyalty, which is why people like to work together for the attainment of project goals. The project managers interested in ensuring project success may practice cronyism with caution by selecting trustworthy team members.

## 5. Limitations and Future Directions

Like other studies, this study has a few limitations that should be considered cautiously while making further examinations. The main reason for the limited sample size was the reluctance on the part of some employees to respond to the questionnaires, because of the fear that if their managers somehow found their answers, they might be penalized, despite being assured of the confidentiality of their responses. Since the IT sector demands close interaction between team leaders and members, the IT sector was selected for the current study. However, further research may be conducted in other work environments encouraging teamwork such as the health sector. 

Moreover, a detailed examination is required to further probe into the relationship between toxic leaders and project success in the presence of cronyism, and to investigate whether toxicity promotes cronyism, or cronyism promotes toxicity. Moreover, the effects of other leadership styles can also be examined to enhance the existing research framework. 

## 6. Conclusions

The high level of social exchange that takes place between toxic leaders and their cronies encourages teamwork. However, toxicity no doubt decreases success in IT projects. Cronyism likely promotes team building and knowledge sharing, thus reducing the toxicity of leaders and turning projects into successes. Cronyism is a soft form of criminal conspiracy but a vital source of networking [23,31] that promotes exchanges among project members and leads to project success.

## Figures and Tables

**Table 1:** Demographic Information, *n* = 240.

Variables	Category	Frequency	Percentage
Gender	Male	165	68.8
	Female	75	31.2
Age (years)	26–35	88	36.7
	36–45	101	42.1
	46–55	51	21.2
Education	Below graduation	38	15.8
	Graduation	44	18.3
	Masters	158	65.9
Experience (years)	Less than 1	71	29.6
	1–5	93	38.8
	6–10	33	13.7
	11–15	23	9.6
	15 and more	20	8.3

Source: Field Data.

**Table 2:** Factor Loading, Composite Reliability, and AVE.

Construct	Loading	C.R.	AVE
Toxic leadership		0.975	0.770
TL1	0.764		
TL2	0.792		
TL3	0.862		
TL4	0.799		
TL5	0.775		
TL6	0.883		
TL7	0.766		
TL8	0.789		
TL9	0.766		
TL10	0.876		
TL11	0.832		
TL12	0.763		
TL13	0.761		
TL14	0.855		
TL15	0.783		
Cronyism		0.966	0.727
CR1	0.870		
CR2	0.701		
CR3	0.776		
CR4	0.889		
CR5	0.752		
CR6	0.862		
CR7	0.779		
CR8	0.754		
CR9	0.897		
Project success		0.969	0.744
PS1	0.875		
PS2	0.858		
PS3	0.868		
PS4	0.863		
PS5	0.873		
PS6	0.874		
PS7	0.870		
PS8	0.812		
PS9	0.855		
PS10	0.881		
PS11	0.866		

Source: SamrtPLS results.

**Table 3:** Discriminant Validity.

Constructs	TL	CR	PS
TL	0.877		
CR	0.702	0.852	
PS	0.492	0.521	0.862

Source: SmartPLS results.

**Table 4:** Relationship of constructs.

Relationship	Coefficient	*p*-Value
TL--->PS (direct)	−0.542	0.000
TL--->PS	0.634	0.000
TL--->CR--->PS	0.327	0.000

Source: SmartPLS output.

## Data Availability

The data will be available on request from the corresponding author.

## References

[1] Bilir C., Yafez E. (2021). Project success/failure rates in Turkey. Int. J. Inf. Syst. Proj. Manag..

[2] Dahie A.M., Osman A.A., Omar A.A. (2017). The role of project management in achieving project success: Empirical study from local NGOs in Mogadishu-Somalia. Int. J. Eng. Sci..

[3] De Clercq D., Fatima T., Jahanzeb S. (2022). Cronies, procrastinators, and leaders: A conservation of resources perspective on employees’ responses to organizational cronyism. Eur. J. Work. Organ. Psychol..

[4] Jansson T., Ljung L. (2004). Projektledningsmetodik.

[5] Yang L.R., Huang C.F., Wu K.S. (2011). The association among project manager’s leadership style, teamwork and project success. Int. J. Proj. Manag..

[6] Zaman U., Florez-Perez L., Anjam M., Khwaja M.G., Ul-Huda N. (2022). At the end of the world, turn left: Examining toxic leadership, team silence and success in mega construction projects. Eng. Constr. Archit. Manag..

[7] Zhu F., Wang L., Yu M., Müller R., Sun X. (2019). Transformational leadership and project team members’ silence: The mediating role of feeling trusted. Int. J. Manag. Proj. Bus..

[8] Saleem F., Malik M.I., Malik M.K. (2021). Toxic Leadership and Safety Performance: Does Organizational Commitment act as Stress Moderator?. Cogent Bus. Manag..

[9] Saqib A., Arif M. (2017). Employee silence as mediator in the relationship between toxic leadership behavior and organizational learning. Abasyn J. Soc. Sci..

[10] Indradevi R. (2016). Toxic leadership over the years—A review. Purushartha-J. Manag. Ethics Spiritual..

[11] Whicker M.L. (1996). Toxic Leaders: When Organizations Go Bad.

[12] Vreja L.O., Balan S., Bosca L.C. Toxic leadership. An evolutionary perspective. Proceedings of the International Management Conference.

[13] Ali S., Shahzad F., Hussain I., Yongjian P., Khan M.M., Iqbal Z. (2022). The Outcomes of Organizational Cronyism: A Social Exchange Theory Perspective. Front. Psychol..

[14] Lipman-Blumen J. (2010). Toxic leadership: A conceptual framework. Handbook of Top Management Teams.

[15] Kusy M., Holloway E. (2009). Toxic Workplace: Managing Toxic Personalities and Their Systems of Power.

[16] Green J.E. (2014). Toxic Leadership in Educational Organizations. Educ. Lead. Rev..

[17] Bell R.M. (2017). The Dysfunction Junction: The Impact of Toxic Leadership on Follower Effectiveness. Ph.D. Thesis.

[18] Veldsman T.H., Johnson A.J. (2016). Leadership: Perspectives from the Front Line.

[19] Lipman-Blumen J. (2006). The Allure of Toxic Leaders: Why We Follow Destructive Bosses and Corrupt Politicians—And How We Can Survive Them.

[20] Tekiner M.A., Aydın R. (2016). Analysis of Relationship Between Favoritism and Officer Motivation: Evidence from Turkish Police Force. Inquiry.

[21] Pelletier K.L., Bligh M.C. (2008). The aftermath of organizational corruption: Employee attributions and emotional reactions. J. Bus. Ethics.

[22] Zhou K., Zha H., Song L. (2013). Learning social infectivity in sparse low-rank networks using multi-dimensional hawkes processes. Artif. Intell. Stat..

[23] Khatri N., Tsang E.W., Begley T.M. (2006). Cronyism: A cross-cultural analysis. J. Int. Bus. Stud..

[24] Al-Khouri A.M., Bal J. (2007). Electronic government in the GCC countries. Int. J. Soc. Sci..

[25] Ramos P., Mota C. (2014). Perceptions of success and failure factors in information technology projects: A study from Brazilian companies. Proc. Soc. Behav. Sci..

[26] LeBlanc D.C. (2008). The Relationship between Information Technology Project Manager Personality Type and Project Success. Ph.D. Thesis.

[27] Ekeh P.P. (1974). Social Exchange Theory: The Two Traditions.

[28] Vischer J.C. (2007). The effects of the physical environment on job performance: Towards a theoretical model of workspace stress. Stress Health J. Int. Soc. Investig. Stress.

[29] Wilfong J.D. (2014). Organizational Culture and Information Technology (IT) Project Success and Failure Factors: A Mixed-Methods Study Using the Competing Values Framework and Schein’s Three Levels Approach. Ph.D. Thesis.

[30] Pelletier K.L. (2010). Leader toxicity: An empirical investigation of toxic behavior and rhetoric. Leadership.

[31] Khatri N., Tsang E.W. (2003). Antecedents and consequences of cronyism in organizations. J. Bus. Ethics.

[32] Cropanzano R., Mitchell M.S. (2005). Social exchange theory: An interdisciplinary review. J. Manag..

[33] Amunkete S., Rothmann S. (2015). Authentic leadership, psychological capital, job satisfaction and intention to leave in state-owned enterprises. J. Psychol. Afr..

[34] Shipton H., Sanders K., Atkinson C., Frenkel S. (2016). Sense-giving in health care: The relationship between the HR roles of line managers and employee commitment. Hum. Res. Manag. J..

[35] Padilla A., Hogan R., Kaiser R.B. (2007). The toxic triangle: Destructive leaders, susceptible followers, and conducive environments. Leadersh. Q..

[36] Reed G.E. (2004). Toxic leadership. Millenn. Rev..

[37] Erdogan B., Bauer T.N. (2014). Leader-member exchange (LMX) theory: The relational approach to leadership. The Oxford Handbook of Leadership and Organizations.

[38] Rose K.H. (2013). A Guide to the Project Management Body of Knowledge (PMBOK^®^ Guide)—Fifth Edition. Proj. Manag. J..

[39] Karlsen J.T., Lereim J. (2005). Management of project contingency and allowance. Cost Eng..

[40] Müller R., Jugdev K. (2012). Critical success factors in projects: Pinto, Slevin, and Prescott-the elucidation of project success. Int. J. Manag. Proj. Bus..

[41] Shrnhur A.J., Levy O., Dvir D. (1997). Mapping the dimensions of project success. Proj. Manag. J..

[42] Thite M. (2000). Leadership styles in information technology projects. Int. J. Proj. Manag..

[43] Taherdoost H., Keshavarzsaleh A. (2015). A theoretical review on IT project success/failure factors and evaluating the associated risks. Mathematical and Computational Methods in Electrical Engineering.

[44] Lewis P. (2004). Transforming Economics: Perspectives on the Critical Realist Project.

[45] Appelbaum S.H., Roy-Girard D. (2007). Toxins in the workplace: Affect on organizations and employees. Corp. Gov. Int. J. Bus. Soc..

[46] Hadadian Z., Zarei J. (2016). Relationship between toxic leadership and job stress of knowledge workers. Stud. Bus. Econ..

[47] Davis Q.L. (2016). A Comprehensive Review of Toxic Leadership.

[48] Bagi S. (2013). When leaders burn out: The causes, costs and prevention of burnout among leaders’. Interdiscip. Perspect. Int. Leadersh..

[49] Sutton J.P., Pae H.A., Bausmith S.C., O’Connor D.M. (2001). Project CREATE: State-wide partnership for producing highly qualified special education teachers. Publ. Law.

[50] Schmidt A.A. (2008). Development and Validation of the Toxic Leadership Scale. Ph.D. Thesis.

[51] Veldsman T.H. (2016). How toxic leaders destroy people as well as organisations. The Conversation.

[52] Priest N., Williams D.R., Major B., Dovidio J.F., Link B.G. (2018). Racial discrimination and racial disparities in health. The Oxford Handbook of Stigma, Discrimination, and Health.

[53] Mehta S., Maheshwari G.C. (2014). Toxic leadership: Tracing the destructive trail. Int. J. Manag..

[54] Harris K.J., Kacmar K.M., Zivnuska S., Shaw J.D. (2007). The impact of political skill on impression management effectiveness. J. Appl. Psychol..

[55] Aboyassin N.A., Abood N. (2013). The effect of ineffective leadership on individual and organizational performance in Jordanian institutions. Compet. Rev. Int. Bus. J..

[56] Tucker M.F., Bonial R., Vanhove A., Kedharnath U. (2014). Leading across cultures in the human age: An empirical investigation of intercultural competency among global leaders. SpringerPlus.

[57] Aubrey D.W. (2012). The Effect of Toxic Leadership. Strategy Research Project, United States Army War College. https://apps.dtic.mil/sti/pdfs/ADA560645.pdf.

[58] Erdem B., Karataş A. (2015). The effects of cronyism on job satisfaction and intention to quit the job in hotel enterprises: The case of three, four and five star hotels in muğla, turkey. Manas Sos. Araştırmalar Derg..

[59] Arasli H., Tumer M. (2008). Nepotism, Favoritism and Cronyism: A study of their effects on job stress and job satisfaction in the banking industry of north Cyprus. Soc. Behav. Personal. Int. J..

[60] Wong L.C., Kleiner B.H. (1994). Nepotism. Work Study.

[61] Abdalla H.F., Maghrabi A.S., Raggad B.G. (1998). Assessing the perceptions of human resource managers toward nepotism. Int. J. Manpow..

[62] O’Brien K.R., McAbee S.T., Hebl M.R., Rodgers J.R. (2016). The impact of interpersonal discrimination and stress on health and performance for early career STEM academicians. Front. Psychol..

[63] Shaheen S., Bashir S., Khan A.K. (2017). Examining organizational cronyism as an antecedent of workplace deviance in public sector organizations. Public Pers. Manag..

[64] Büte M. (2011). The effects of nepotism and favoritism on employee behaviors and human resources practices: A research on Turkish public banks. TODAĐE’s Rev. Public Adm..

[65] Azam N., Qureshi R. (2018). Khandan and Talluqat as social capital for women’s career advancement in Pakistan: A study of NGO sector in Islamabad. Proceedings of the International Conference on Gender Research, Porto, Portugal, 12–13 April 2018.

[66] Cingoz A., Akilli H.S. A study on examining the relationship among cronyism, self-reported job performance, and organizational trust. Proceedings of the 2015 WEI International Academic Conference.

[67] Shabbir B., Siddique H. (2017). Impact of Nepotism, Cronyism, and Favoritism on Organizational Performance with a Strong Moderator of Religiosity. Int. J. Sci. Eng. Res..

[68] Asgharian R., Anvari R., Ahmad U.N.U.B., Tehrani A.M. (2015). The mediating effect of job satisfaction on the relationship between workplace friendships and turnover intention in Iran hotel industry. Mediterr. J. Soc. Sci..

[69] Philibin C.A.N., Griffiths C., Byrne G., Horan P., Brady A.M., Begley C. (2010). The role of the public health nurse in a changing society. J. Adv. Nurs..

[70] Leung T.K.P., Bradley R.B. (2020). Ethical cronyism: An insider approach for building guanxi and leveraging business performance in China. Asia Pac. Bus. Rev..

[71] Lai J.Y., Chow C.W., Loi R. (2018). The interactive effect of LMX and LMX differentiation on followers’ job burnout: Evidence from tourism industry in Hong Kong. Int. J. Hum. Res. Manag..

[72] Hayajenh A.F., Maghrabi A.S., Al-Dabbagh T.H. (1994). Research note: Assessing the effect of nepotism on human resource managers. Int. J. Manpow..

[73] Turhan M. (2014). Organizational cronyism: A scale development and validation from the perspective of teachers. J. Bus. Ethics.

[74] Nelson R.R. (2007). IT project management: Infamous failures, classic mistakes, and best practices. MIS Q. Exec..

[75] Hair J.F., Ringle C.M., Sarstedt M. (2012). Partial least squares: The better approach to structural equation modeling?. Long Range Plan..

[76] Fornell C., Larcker D.F. (1981). Structural equation models with unobservable variables and measurement error: Algebra and statistics. Qual. Res..

[77] Ehsan I. (2020). Impact of Abusive Leadership on Project Success with Mediating Role of Workplace Deviance and Moderating Role of Agreeableness. Ph.D. Thesis.

[78] Abbas M., Ali R. (2021). Transformational versus transactional leadership styles and project success: A meta-analytic review. Eur. Manag. J..

[79] Ahmed E.M.A. (2021). Leadership and organizational distress: Review of literature. Int. J. Res. Bus. Soc. Sci..

[80] Labrague L.J. (2021). Influence of nurse managers’ toxic leadership behaviours on nurse-reported adverse events and quality of care. J. Nurs. Manag..

[81] Kurtulmuş B.E. (2020). Toxic leadership and workplace bullying: The role of followers and possible coping strategies. The Palgrave Handbook of Workplace Well-Being.

[82] Pimenta F.X.E. (2022). Toxicity at Work Place: Coping Strategies to Reduce Toxins at Work Place. ECS Trans..

[83] Jawahar I.M., Bilal A.R., Fatima T., Mohammed Z.J. (2021). Does organizational cronyism undermine social capital? Testing the mediating role of workplace ostracism and the moderating role of workplace incivility. Career Dev. Int..

[84] Mergen A., Ozbilgin M.F. (2021). Understanding the followers of toxic leaders: Toxicillusiono and personal uncertainty. Int. J. Manag. Rev..

[85] Islam N. (2004). Sifarish, sycophants, power and collectivism: Administrative culture in Pakistan. Int. Rev. Adm. Sci..

